# The regulatory role of differential microRNA expressions on cellular inflammatory factors IL‐6 and IL‐10 in *Echinococcus granulosus*‐induced anaphylaxis

**DOI:** 10.1002/iid3.961

**Published:** 2023-08-08

**Authors:** Chun‐sheng Wang, Tao Yu, Xilizhati Kulaixi, Jing‐ru Zhou, Xianyidan Abulajiang, Jia‐ling Wang, Si‐jia Wang, Jian‐rong Ye

**Affiliations:** ^1^ Department of Anesthesiology The First Affiliated of Xinjiang Medical University Urumqi Xinjiang China; ^2^ Shandong Institute of Parasitic Diseases Shandong First Medical University (Shandong Academy of Medical Sciences) Jinan Shandong China

**Keywords:** echinococcosis‐induced anaphylaxis, IL‐6, IL‐10, inflammatory reactions, miRNA

## Abstract

**Objective:**

To determine the pathogenesis and molecular targets of anaphylaxis caused by hydatid cyst fluid leakage.

**Methods:**

First, Balb/c mice were infected with *Echinococcus granulosus*, and then the anaphylaxis model was developed. The mice were separated into: anaphylaxis caused by the cystic echinococcosis group (ANPC), the cystic echinococcosis without anaphylaxis group (CE group), and the normal control group (CTRL). Following this, the spleen tissue was collected for microRNA (miRNA) sequencing. Using bioinformatics analysis, differentially expressed miRNAs (DEMs) were identified. Then, through the use of protein–protein interaction (PPI) networks, the key target genes for miRNA regulation associated with echinococcosis‐induced anaphylaxis were identified.

**Results:**

ANPC and CE groups have 29 and 39 DEMs compared to the CTRL group, respectively. Based on these 25 DEMs, interactions between miRNA and mRNA were screened, and 174 potential target genes were identified. We performed gene ontology (GO) function and Kyoto Encyclopedia of Genes and Genomes pathway enrichment analysis on these 174 target genes, and the results revealed that the three pathways with the highest enrichment were the PI3K‐Akt signaling pathway, FoxO signaling pathway, and Focal adhesion. The interaction analysis of PPI and miRNA‐hub gene networks revealed that interleukin 6 (IL‐6) was regulated by miR‐146a‐5p and miR‐149‐5p, while IL‐10 was regulated by miR‐29b‐3p and miR‐29c‐3. Using reverse transcription polymerase chain reaction, we found that the miRNAs regulating IL‐6 and IL‐10 were significantly upregulated in the ANPC group, and there are three pathways involved in that process: Pathways of PI3K‐Akt signaling, FoxO signaling, and Focal adhesion. IL‐6 and IL‐10 play an important role in cellular pyroptosis and apoptosis. Therefore, the aforementioned results provide significant reference value for elucidating the mechanism of cellular pyroptosis and apoptosis in echinococcosis‐induced anaphylaxis, and for formulating tissue and organ protection strategies for patients with cystic echinococcosis when anaphylaxis is triggered by hydatid cyst rupture.

## 1 INTRODUCTION

Echinococcosis, a severe zoonotic disease caused by *Echinococcus granulosus* parasitizing humans and certain animal hosts, is a chronic global disease that can lead to significant veterinary, medical, and economic issues.^1^ Cystic echinococcosis is a disease brought on by the ingestion of eggs of *E. granulosus*. Many provinces in China are affected by cystic echinococcosis, with Xinjiang, Inner Mongolia, Sichuan, Gansu, Ningxia, and Tibet having the highest prevalence of patients with cystic echinococcosis. The disease has impeded the growth of the local economy and animal husbandry.^2^ Cystic echinococcosis is highly prevalent in Xinjiang, with a 2%–4% mortality rate. In case of improper treatment, the mortality rate can be higher.^1^

In preliminary clinical practice, it has been determined that anaphylaxis resulting from surgery, trauma, and spontaneous cyst rupture are severe complications of the disease. This aggressive complication may result in severe consequences or even death if treated improperly.^3^ Currently, it is believed that anaphylaxis is a multifactorial polygenetic, immune disease caused by a large number of minor genes and environmental factors.^4^ MicroRNAs (miRNAs) are involved in the production and differentiation of immune cells and have a close relationship with immune system diseases.^5^ miRNA sequencing is a crucial technique that can simultaneously quantify the miRNA expression levels of a large number of tissues to efficiently identify differentially expressed miRNAs (DEMs). Additionally, it can be used to simultaneously analyze and predict target genes that are regulated by DEMs. The expressions of interleukin 10 (IL‐10) and IL‐6 were significantly increased during anaphylaxis in patients with cystic echinococcosis, according to previous studies,^6^, ^7^ and IL‐10 and IL‐6 play a crucial role in the occurrence and development of a variety of inflammatory diseases.^8^, ^9^ At the same time, IL‐10 is closely associated with the impairment of cellular function caused by pyroptosis and apoptosis.^10^, ^11^ Additionally, IL‐6 has the same effects in the two aspects mentioned above.^12^ Therefore, bioinformatics analysis can be used to determine the DEMs and regulatory targets associated with IL 10 and IL 6. As a result, it provides significant reference value for elucidating the mechanisms underlying the effects of miRNAs expressed in echinococcosis‐induced anaphylaxis on cellular pyroptosis and apoptosis, and for formulating tissue and organ protection strategies for patients with cystic echinococcosis when anaphylaxis is triggered by hydatid cyst rupture.

## 2 MATERIALS AND METHODS

### 2.1 Experimental animals, crude hydatid cyst fluid, and *E. granulosus*

#### 2.1.1 Experimental animals

A total of 18 female BALB/c mice, aged 4–8 weeks and weighing (20 ± 2) g, were purchased from the Laboratory Animal Center of the First Affiliated Hospital of Xinjiang Medical University.

#### 2.1.2 Crude hydatid cyst fluid

The liver of sheep containing cysts was collected from the slaughterhouse in Urumqi, Xinjiang. After cleaning and drying the liver, the cyst fluid containing protoscoleces was collected and naturally precipitated. The supernatant was obtained, and the endotoxin was removed. The lipopolysaccharide (LPS) concentration was below 1.2 endotoxin units/mg. Using the BCA protein kit, the protein level was determined to be greater than 2815.2 µg/mL. The sample was kept in a refrigerator at −80°C. The sample was taken out 24 h before anaphylaxis and stored at 4°C.

#### 2.1.3 Echinococcus granulosus

The cystic fluid was digested with 1% pepsin. The cyst fluid was aspirated slowly after protoscoleces naturally settled to the bottom. Protoscoleces were inoculated at a culture density of 2000/mL in RPMI 1640 medium and incubated at 37°C for 40 days in an incubator with 5% CO_2_ and a constant temperature of 37°C.

### 2.2 Animal grouping and model establishment

#### 2.2.1 Experimental grouping

There were six mice in the cystic echinococcosis without anaphylaxis (CE) group (establishment of the echinococcosis‐infected model, with no anaphylaxis after saline injection), six in the anaphylaxis caused by cystic echinococcosis (ANPC) group (establishment of the echinococcosis‐infected model, with anaphylaxis after hydatid cyst fluid injection), and six in the normal control (CTRL) group (not infected with echinococcosis, injected with normal saline).

#### 2.2.2 Animal modeling

The microcapsules were inoculated via intraperitoneal injection to infect BALB/c mice (50/mouse) to establish the *E. granulosus* infection model of BALB/c mice. An equal amount of normal saline was injected in the CTRL group. Six months after infection, crude cystic fluid of sheep‐derived *E. granulosus* was injected intraperitoneally at a dosage of 0.1 mL/10 g mouse body weight to induce anaphylaxis. An equal amount of normal saline was injected in the CTRL group. After anaphylaxis, the mice were scored based on a symptom score table (refer to the classic reaction symptom table of equine serum‐induced mice anaphylactic shock), and their anus temperature was measured every 5 min. After 1 h of anaphylaxis, the epicanthal venous blood was extracted, and the spleen was collected to prepare single‐cell suspension.

Refer to the classic reaction symptom tables of equine serum‐induced anaphylactic shock in mice.^11^

### 2.3 miRNA sequencing

Three mice were collected from the CTRL, CE, and ANPC groups, respectively. The spleen tissue was quick‐frozen with liquid nitrogen and then stored at −80°C. The sequencing was performed using the Illumina HiSeq. 2000 platform. RAW reading began with data cleansing, which included the removal of adapters, inferior tags, and several contaminants from 50 NT tags. The label length distribution was then summarized. The RNA‐seq data set was visualized using the Integrative Genome Observer.

### 2.4 Reverse transcription polymerase chain reaction verification

We analyzed the relative expression of key mRNAs in mice infected with *E. granulosus* by summarizing the pathway enrichment analysis network with quantitative reverse transcription polymerase chain reaction (qRT‐PCR) and verified the previous research results. GAPDH was used as an internal control, and the cycle threshold (Ct) value was normalized to determine the gene expression level. The $2^{‐{\mathrm{\Delta \Delta C}}_{t}}$ method was used to calculate the expression of relevant genes, with three replicates per group to ensure quantification accuracy.

### 2.5 Data analysis

#### 2.5.1 DEM screening and Venn analysis

The edgeR package in R software was used to examine the relationships between the differential miRNA expressions of the ANPC and CE groups, and the CTRL group. *p* < .05 was chosen as the threshold for identifying DEMs. Overlapping miRNAs in both datasets were screened using Venn analysis and the VennR package.

#### 2.5.2 Target gene prediction and miRNA pathway analysis

RAVID v2.0 is an experimentally validated database of miRNA target interactions used to predict potential DEM target genes. The miRNACancerMAP database was adopted for miRNA pathway analysis.

#### 2.5.3 Gene ontology and Kyoto Encyclopedia of Genes and Genomes pathway analysis

Using the gene ontology (GO) and Kyoto Encyclopedia of Genes and Genomes (KEGG) databases, the data were processed with the DAVID online tool to perform a functional and pathway analysis (https://david.ncifcrf.gov/) of the predicted target genes of 25 DEMs. Values of *p* < .05 were considered significant.

#### 2.5.4 Construction of target gene PPI and miRNA‐hub gene networks

To determine the functional relationship between target genes that upregulate and downregulate DEM, we uploaded the target gene data to the STRING database. An interaction with a comprehensive score >0.4 was considered significant. Cytoscape software (version 3.6.0) was used to analyze the highly connected (hub) genes in protein–protein interaction (PPI) networks. After identifying the hub‐dem and hub‐target genes, the cell landscape was used to visualize the obtained miRNA gene network.

#### 2.5.5 Statistical analysis

GraphPad Prism 7.0 software was used for plotting and statistical analysis. The measurement data are expressed as the mean ± standard deviation (*X* ± SD), and a one‐way analysis of variance was used for between‐group comparisons. The significance level is *α* = .05.

### 2.6 Ethical approval

Regarding the welfare of experimental animals, we followed the rules and systems of the Laboratory Animal Center of the First Affiliated Hospital of Xinjiang Medical University in this study. The ethical approval number is IACUC20170315‐04.

## 3 RESULTS

### 3.1 Screening and identification of DEMs and target genes

**Figure 1:**
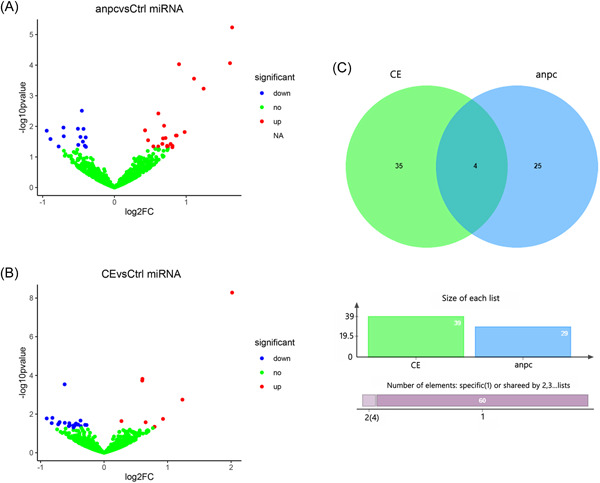
Differentially expressed miRNAs (DEMs). (A) DEM volcano map of ANPC versus CTRL difference; (B) DEM volcano map of CE versus CTRL difference; (C) Venn map of difference of DEM.

To determine the DEMs in echinococcosis‐induced anaphylaxis, miRNA expression data of samples from the ANPC, CE, and CTRL groups were obtained by miRNA sequencing, and then standardized using the limma package in R software. According to the defined threshold (*p* < .05 Figure 1A,B), there were 29 and 39 DEMs in the ANPC and CE groups, respectively, as compared with the CTRL group. Subsequently, the Venn plot analysis identified a total of 4 DEMs between these two data sets (Figure 1C), whereas 25 DEMs were specifically expressed in echinococcosis‐induced anaphylaxis. Based on these 25 DEMs, the miRNA target gene interactions were evaluated using the experimentally verified RAID V2.0 database, and the interaction relationships with a miRNA–mRNA interaction score greater than 0.5 were screened. A total of 174 potential target genes were identified.

### 3.2 miRNAs target genes are involved in function and pathways

**Figure 2:**
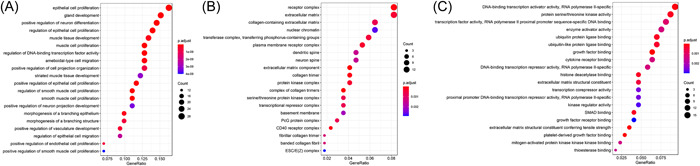
Functions associated with target genes for miRNAs. A: biological processes of target genes; B: cellular components of target genes; C: molecular functions of target genes.

GO functional annotation analysis revealed that the most frequently observed terms for the targets of 174 miRNAs were “epithelial cell proliferation” and “regulation of DNA‐binding transcription factor activity” in the category of “biological processes (BP)” (Figure 2A), “collagen trimer complex” and “CD40 receptor complex” in the category of cell components (CC) (Figure 2B), AND “growth factor binding” in the category of molecular function (MF) (Figure 2C). The three most significantly enriched KEGG pathways were the PI3K‐Akt signaling pathway, FoxO signaling pathway, and Focal adhesion (Table 1).

**Table 1:** The KEGG pathways involved in the miRNAs target genes.

Pathways	Number of enriched genes	Enriched genes were a percentage of target genes	*P*‐Value
Pathways in cancer	33	19.07514	8.36E‐18
PI3K‐Akt signaling pathway	27	15.60694	1.78E‐13
Prostate cancer	14	8.092486	7.36E‐11
Small cell lung cancer	13	7.514451	6.22E‐10
Focal adhesion	18	10.40462	9.83E‐10
Proteoglycans in cancer	17	9.82659	5.87E‐09
FoxO signaling pathway	14	8.092486	1.49E‐08
Epstein‐Barr virus infection	14	8.092486	1.78E‐08
HTLV‐I infection	17	9.82659	4.37E‐07
MicroRNAs in cancer	17	9.82659	4.37E‐07

### 3.3 Construction of target gene PPI and miRNA‐hub gene networks

**Figure 3:**
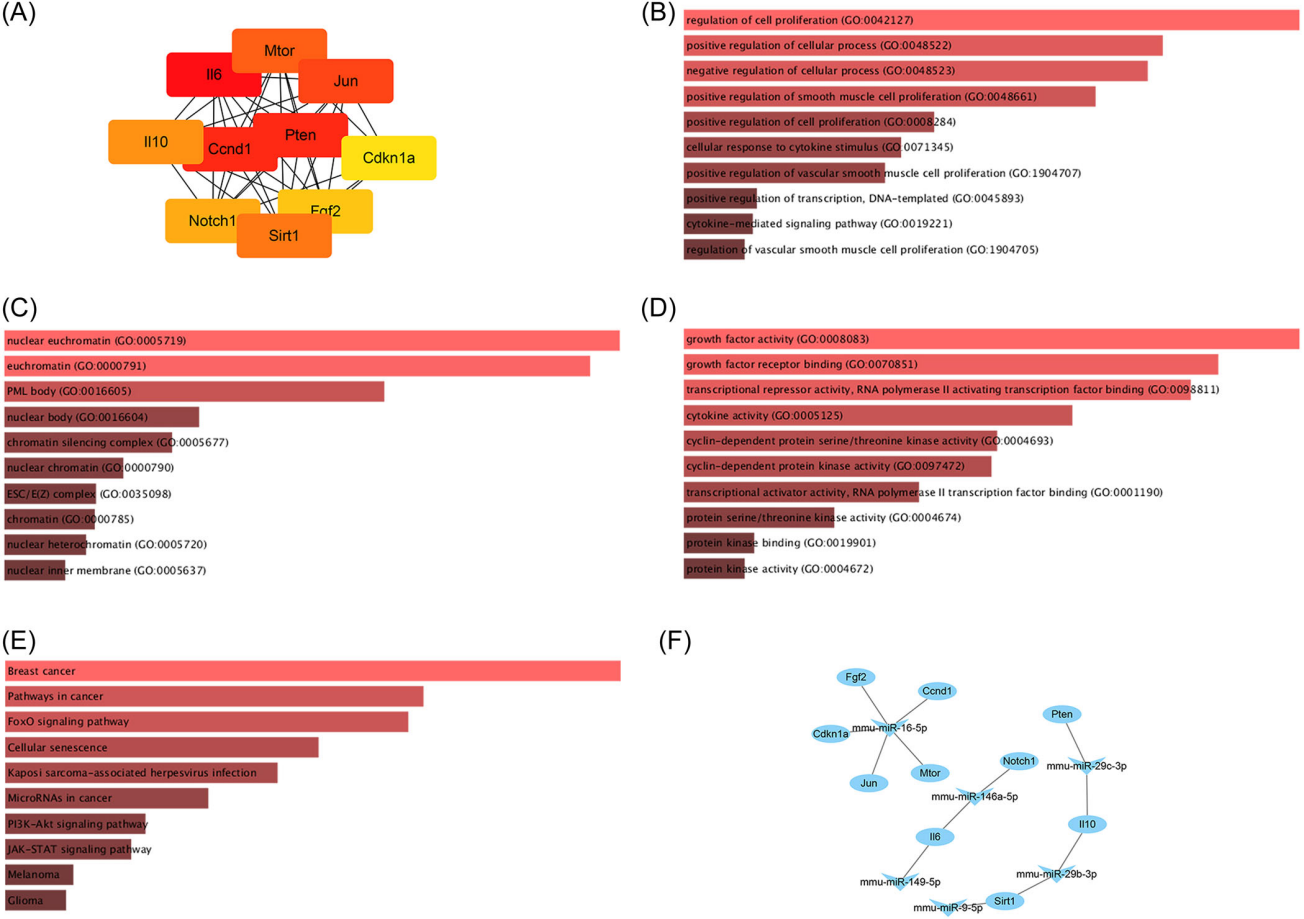
Construction of the target gene PPI and miRNA‐hub gene network. (A) Hub genes predicted; (B) biological processes of hub genes; (C) cellular components of Chub genes; (D) molecular functions of hub genes; (E) hub genes involved in the KEGG pathway; and (F) regulation of hub gene on IL‐10 and IL‐6. KEGG, Kyoto Encyclopedia of Genes and Genomes; IL, interleukin; PPI, protein–protein interaction.

A PPI network was constructed to identify the hub gene among the targets of the 25 DEMs. The predicted hub genes for the 25 identified miRNAs are IL‐6, Pten, Ccnd1, Jun, Mtor, Sirt1, IL‐10, Notch1, Fgf2, and Cdkn1a (Figure 3A). We also performed GO and KEGG analyses on these gene clusters to verify their potential significance in the development of echinococcosis‐induced anaphylaxis. GO analysis revealed that these gene clusters were significantly enriched in different functions, including “regulation of cellular proliferation” and “positive regulation of cellular processes” in the biological process (BP) classification (Figure 3B), “nuclear euchromatin” and “chromatin silencing complex” in the cellular component (CC) category (Figure 3C), AND “growth factor activity” and “transcription inhibitor activity, RNA polymerase II activated transcription factor binding” in the molecular function (MF) category (Figure 3D). Additionally, these genes are enriched in the PI3K‐Akt signaling pathway, the FoxO signaling pathway, and other relevant signaling pathways (Figure 3E). Using Cytoscape software, the miRNA‐hub gene network was subsequently constructed. Based on the results of interaction analysis, 5 of the 10 hub genes targeted by miRNAs, may be regulated by miR‐16‐5p (Fgf2, Cdkn1a, Ccnd1, Mtor, and Jun; Figure 3F), while IL‐6 may be regulated by miR‐146a‐5p and miR‐149‐5p (Figure 3F), and IL‐10 may be regulated by miR‐29b‐3p and miR‐29c‐3p (Figure 3F).

### 3.4 Detection of mRNA expression of target genes IL‐6 and IL‐10 by RT‐PCR

**Figure 4:**
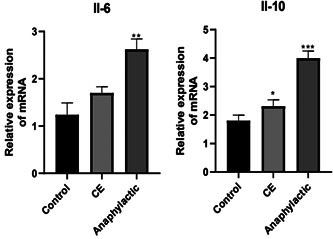
The mRNA expression of IL‐6 and IL10 in mice. * Compared to the control group, *p < .05; **p < .01; ***p < .001. IL, interleukin.

The results showed that the levels of IL‐6 in the CTRL, CE, and ANPC groups were 1.30 ± 0.56, 1.68 ± 0.33, and 2.64 ± 0.45 pg/mL, respectively. Compared to the CTRL group, both the CE and ANPC groups had significantly increased IL 6 levels, with the degree of increase in the ANPC group being particularly significant (*p* < .01). The IL‐10 levels in the CTRL, CE, and ANPC groups were 1.89 ± 0.23, 2.28 ± 0.33, and 3.95 ± 0.35, respectively. As compared with the CTRL group, the IL‐10 level was significantly elevated in both the CE and ANPC groups (*p* < .05, *p* < .001; Figure 4).

## 4 DISCUSSION

Traditionally, hydatid cyst fluid leakage‐induced anaphylaxis has been considered predominantly sporadic, with the cause possibly being genetic differences in clinical phenotypes.^13^ All of these indicate that a significant gene regulatory mechanism is involved in the pathogenesis of hydatid cyst fluid leakage‐induced anaphylaxis. At present, molecular biology research on hydatid cyst fluid leakage‐induced anaphylaxis has been widely carried out, but is usually limited to the detection of single or multiple indicators. However, hydatid cyst fluid leakage‐induced anaphylaxis may be the result of a complex process involving numerous genes, multiple links, and various mechanisms.

miRNA is a class of short‐stranded noncoding RNA with a length between 18 and 22 nt. After cleavage by the Drosha and Dicer enzymes, mature miRNAs are integrated into RNA‐induced silencing complexes and bind to the mRNA 3′ untranslated region of target genes to promote the degradation of target genes or inhibit their translation.^14^ miRNA plays a key role in regulating eukaryotic gene expression and participates in biological processes such as cellular proliferation, differentiation, development, metabolism, and apoptosis.^15^ In parasites, miRNAs not only regulate the growth and development of the worms, but also induce changes in the host miRNAs to either promote or inhibit parasitic infections.^16^

Among the 25 specifically expressed miRNAs in the ANPC group, the target genes of miR‐146a‐5p, miR‐29b‐3p, miR‐29c‐3p, miR‐149‐5p, and miR‐16‐5p were significantly correlated with inflammatory responses. There are two miR‐146 genes that are evolutionarily conserved in mice, namely, miR‐146a and miR‐146b. Current studies suggest that miR‐146a and miR‐146b affect both microbial infection and pro‐inflammatory cytokine synthesis.^17^ However, after persistent infection with *Toxoplasma gondii*, miR‐146a expression was significantly upregulated in nonhematopoietic cells.^18^ After *E. multilocularis* infection, miR‐146a‐5p in the peripheral blood serum of mice was also significantly upregulated.^19^ Studies have shown that miR‐149‐5p inhibits inflammation by regulating signals such as toll‐like receptors (TLRs) and signal transducers and activators of transduction 3 (STAT3), and is involved in the progression of various diseases; in addition, it is associated with innate immune dysfunction.^20^

Seasonal urban particulate matter (PM) can increase the expression of three miRNAs (miR‐146a, miR‐1246, and miR‐29c), which are associated with inflammation, oxidative stress, and epigenetics. In addition, studies have also demonstrated that exposure of airway epithelial cells and alveolar macrophages to PM significantly upregulates miR‐29b‐3p, which promotes the occurrence and development of inflammation. Additionally, miR‐29b‐3p can directly target C1QTNF6, thereby inhibiting the activation of the anti‐inflammatory AMPK signaling pathway. These results suggest that miR‐29b‐3p and miR‐29c‐3p regulate the inflammatory response.^21^

However, their biological functions and mechanisms in *E. granulosus* infection and subsequent anaphylaxis remain unknown.

Usually, the allergic reaction caused by *Echinococcus* capsulatus cyst fluid is a systemic validated reaction whose target organs are usually the lungs, liver, and other organs.^22^, ^23^ The spleen, as one of the important immune organs in the human body, has also received attention from researchers regarding splenic injury caused by *Echinococcus* capsulatus cyst fluid, and some studies have shown that both encapsulation can cause splenic injury in buffaloes and patients,^24^, ^25^ but studies on splenic allergic reactions caused by *Echinococcus* capsulatus cyst fluid are still rare, so we would like to investigate what kind of differential miRNAs are expressed in the spleen to participate in the occurrence of systemic inflammatory response, and whether such differential miRNAs are involved in some mechanisms of organ damage related to inflammatory response.

The PPI network interaction diagram revealed that IL‐6 was regulated by miR‐146a‐5p and miR‐149‐5p, while IL‐10 was regulated by miR‐29b‐3p and miR‐29c‐3p. Additionally, RT‐PCR verification revealed that the expressions of IL‐6 and IL‐10 were significantly upregulated in the spleen tissues of the ANPC group. IL‐6 is a cytokine secreted by Th2 cells that can enhance the role of IL‐4 in promoting IgE synthesis and participate in the immune response and immune regulation of the body.^26^, ^27^ When IL‐6 expression is inhibited, cellular apoptosis is also inhibited.^28^ IL‐10 has pleiotropic effects on the regulation of immune function. It is derived primarily from T cells, allowing T cells to remain active while regulating T cells to maintain homeostasis in the internal environment. IL‐10 can also promote pro‐inflammatory CD8^+^ T cell activity, and promote an inflammatory response in IL‐10‐positive CD4^+^ cells, in addition to its expected anti‐inflammatory properties in autoimmune diseases.^29^ In conclusion, IL‐6 and IL‐10 exhibited pro‐inflammatory effects during hydatid cyst fluid‐induced anaphylaxis in mice.

The majority of the 174 target genes regulated by differential miRNAs were enriched in three pathways, that is, the PI3K‐Akt signaling pathway, the FoxO signaling pathway, and the Focal adhesion signaling pathway, based on the KEGG signaling pathway results. miR‐16‐5p regulates 5 target genes (Fgf2, Cdkn1a, Ccnd1, Mtor, and Jun) among the 10 hub genes targeted by miRNAs. These five target genes are also associated with the PI3K‐Akt signaling pathway. In patients with cystic echinococcosis, the levels of TLR2 and TLR4 in blood are significantly increased.^30^ TLR2 and TLR4 can induce the production of a variety of pro‐inflammatory cytokines and promote inflammatory responses in islets, fat, liver, and kidney tissues, ultimately leading to apoptosis of cells, and tissue and organ function damage.^31^ Through the PI3K‐Akt signaling pathway, TLR4 activation can lead to cellular pyroptosis, resulting in tissue and organ damage.^32^ After invasion of pathogenic microorganisms into the body, activation of TLR2 mobilizes neutrophils via FOXO1, which contributes to the elimination of pathogens from the body.^33^ However, an increase in neutrophils would result in pyroptosis by secreting IL‐1β.^34^ Previous research has also documented the activation and increase in neutrophils.^6^ In the focal adhesion signaling pathway, focal adhesion kinase (FAK) is an essential part of the basal cell adhesion, which is called the focal adhesion complex. Loss of matrix adhesion and apoptosis of endothelial cells would result from disruption of FAK signaling. Through the PI3K/Akt signaling pathway, basic FAK activity maintains adhesion between cells and integrins in endothelial cells that have not been stimulated by pathogens. When endothelial cells detach from the underlying extracellular matrix, apoptosis occurs, resulting in endothelial cell damage due to TLR4 activation and inflammatory factors such as TNF‐α.^35^ A significant increase in inflammatory factors such as TNF‐α has also been observed in previous studies,^6^ which induce cellular apoptosis^36^ and pyroptosis^37^ while promoting an inflammatory response in cells and tissues. FAK tyrosine phosphorylation can inhibit cellular apoptosis through the PI3K signaling pathway.^35^ Therefore, the interaction between the focal adhesion signaling pathway and the PI3K‐Akt signaling pathway is likely to play a protective role in the function of tissues and organs in hydatid cyst fluid‐induced anaphylaxis. Nevertheless, further research is required to confirm this effect.

## 5 CONCLUSION

In this study, miRNA sequencing analysis revealed that miR‐146a‐5p, miR‐29b‐3p, miR‐29c‐3p, miR‐149‐5p, and miR‐16‐5p play a crucial role in hydatid cyst fluid‐induced anaphylaxis. In response to anaphylaxis, these miRNAs regulate the expression of the cytokines IL‐6 and IL‐10 and participate in signaling pathways such as PI3K‐Akt. These 5 miRNAs‐miR‐146a‐5p, miR‐29b‐3p, miR‐29c‐3p, miR‐149‐5p, and miR‐16‐5p can therefore be considered as regulatory genes for hydatid cyst fluid‐induced anaphylaxis. Thus, it provides a significant reference value for elucidating the mechanisms underlying cellular pyroptosis and apoptosis in echinococcosis‐induced anaphylaxis, as well as in formulating tissue and organ protection strategies for patients with cystic echinococcosis when anaphylaxis is triggered by hydatid cyst rupture.

## AUTHOR CONTRIBUTIONS

**Chun‐sheng Wang**: Conceptualization; data curation; project administration; software; supervision; writing—original draft. **Tao Yu**: Data curation; formal analysis; investigation; validation; visualization; writing—original draft. **Xilizhati Kulaixi**: Data curation; formal analysis; methodology; validation; visualization; writing—review and editing. **Jing‐ru Zhou**: Investigation; methodology; project administration; software; validation. **Xianyidan Abulajiang**: Data curation; supervision; validation; visualization; writing—review and editing. **Jia‐ling Wang**: Data curation; formal analysis; methodology; project administration; validation; writing—review and editing. **Si‐jia Wang**: Data curation; formal analysis; supervision; validation; visualization; writing—review and editing. **Jian‐rong Ye**: Funding acquisition; project administration; writing—review and editing.

## CONFLICT OF INTEREST STATEMENT

The authors declare no conflict of interest.

## Data Availability

All data generated or analyzed during this study are included in this article. Further inquiries can be directed to the corresponding author.
